# Data for Equity: Can Linked Administrative Data Inform Pathways to More Equitable Child Health?

**DOI:** 10.5694/mja2.70149

**Published:** 2026-03-02

**Authors:** Sarah Gray, Shuaijun Guo, Meredith O'Connor, Elodie O'Connor, Katrina Williams, Hannah Badland, Susan Woolfenden, Josie Dickerson, Gerry Redmond, Marnie Downes, Sharon R. Goldfeld

**Affiliations:** ^1^ Centre for Community Child Health Murdoch Children's Research Institute Melbourne Victoria Australia; ^2^ University of Melbourne Melbourne Victoria Australia; ^3^ Melbourne Children's LifeCourse Initiative, Murdoch Children's Research Institute Melbourne Victoria Australia; ^4^ Monash University Melbourne Victoria Australia; ^5^ Murdoch Children's Research Institute Melbourne Victoria Australia; ^6^ Social Equity Research Centre, RMIT University Melbourne Victoria Australia; ^7^ University of Sydney Sydney New South Wales Australia; ^8^ Sydney Institute for Women, Children and Their Families, Sydney Local Health District Sydney New South Wales Australia; ^9^ Born in Bradford, Bradford Institute for Health Research, Bradford Teaching Hospitals NHS Foundation Trust Bradford England UK; ^10^ Flinders University Adelaide South Australia Australia

**Keywords:** administrative data, child health, health equity

## Abstract

Child health inequities remain a persistent challenge, with well‐described long‐term consequences. Advances in cross‐sector administrative data linkage and causal inference methods offer powerful opportunities to transform data into evidence for addressing inequities. This article explores how linked administrative data support timely, precise, agile and coordinated policy responses and monitor their impact. We outline conditions needed to realise this potential, including sustained cross‐sector data infrastructure, analytic capability and increased efforts to translate evidence into action. We argue linked administrative data can inform pathways to more equitable child health and, with investment, help deliver on lasting returns for children, families and society.

## 1 Introduction

Child health inequities carry substantial short‐ and long‐term costs for individuals and society across health, education and welfare systems [1]. Governments globally are increasingly prioritising early childhood and cross‐sector strategies to promote equitable child health, as demonstrated through Australia's *Early Years Strategy* and the *National Children's Mental Health and Wellbeing Strategy* [1, 2, 3, 4]. Yet data from the Australian Early Development Census (AEDC) show health and developmental inequities at school entry have persisted over the past 15 years [5]. Progress has been hampered by uncertainty about where and how to intervene, challenges translating evidence into action, and the cost and complexity of large‐scale evaluation. We argue that linked administrative data offer a powerful, low‐cost tool to generate scalable insights to inform timely and equitable policies.

Administrative data—routinely collected by government agencies across sectors such as health, education and social care—are transforming the policy and research landscape [6]. Advances in computing power and linkage methods now enable high‐quality, regularly updated longitudinal data assets, such as the Person Level Integrated Data Asset (PLIDA) [7], the Life Course Data Initiative [8] and the National Disability Data Asset [9], that provide a life course view of children's health, development and service use. These developments are supported by the Office of the National Data Commissioner to promote safe sharing of government data for public benefit and research innovation [10]. Together with growing societal expectations for transparency and accountability, this is driving a shift toward data‐informed decision‐making [6].

Current policy and data environments create a unique opportunity to align linked data, advanced analytics and policy effort to advance child health equity. Realising this potential requires more than technical capability and access; it demands a coordinated, equity‐focused system that turns data into timely, actionable evidence. This article outlines what is needed to ensure that linked administrative data can support policy to transform children's health for generations.

## 2 Using Linked Administrative Data to Advance Child Health Equity

The opportunities and challenges of using linked administrative data are well documented (Table 1) [6, 7, 11, 12], but applying these data to address child health inequities is complex. These inequities are shaped from birth by the social determinants of health—the conditions in which children live, learn and grow—which span multiple sectors (e.g., education, social services, taxation, housing and justice) [1]. Addressing child health inequities requires stacking complementary interventions across sectors, particularly during the critical early years [1]. In Australia, priority populations, including First Nations children, children with disabilities, those in out‐of‐home care, children experiencing socio‐economic disadvantage and those from ethnically minoritised or refugee communities, are disproportionately affected by inequities due to structural and systemic factors, including racism [13]. Integrated cross‐sector data, disaggregated for priority populations, are therefore essential to identify who is most affected, monitor reach and ensure responses do not inadvertently widen gaps [11]. Recent work [14, 15] using a longitudinal child‐centred PLIDA data asset highlights both the potential and current limitations (e.g., missing, incomplete or low‐quality indicators) of linked administrative data for capturing early drivers of inequity and informing equity‐focused policy action (Table 2).

**TABLE 1:** Opportunities and challenges for researchers using linked administrative data for research.

	Description
The opportunity	
Breadth of data	Coverage of large populations and inclusion of small or priority populations who have historically been excluded or marginalised by traditional data collection efforts. Captures structural determinants.
Routinely updated data	Data are continually updated, providing a dynamic and timely source for policy‐relevant research.
Analytic insights	Data enable complex, longitudinal and predictive analyses that can offer insights at both individual and population levels, particularly useful for examining trends and impacts over time.
Cost‐efficient	Data are collected for administrative purposes and do not involve additional data collection costs, offering a cost‐effective and efficient alternative to traditional data collection (e.g., clinical trials, surveys).
Objective measures	Data can be less subjective and more accurate than survey data, especially for objective measures like income and health service usage.
Policy relevance	Data are inherently policy‐relevant as they are generated through routine service delivery and are often linked to policy and reporting needs.
The challenge	
Data quality and completeness	Administrative data are not collected for research purposes, which can lead to missing data, inaccuracies and gaps. Changes in data systems or disruptions can also complicate historical comparisons and analyses.
Biases	Errors in data linkage, selective data collection and variations in data processing can introduce biases, potentially misrepresenting populations.
Governance	Complex legal and regulatory environments can delay or hinder access. Indigenous data governance remains largely unembedded. Limited transparency and community involvement risks undermining trust.
Ethical and privacy concerns	Administrative data use raises concerns about informed consent, de‐identification and the potential for misuse or misinterpretation, especially when working with priority populations, which can exacerbate existing inequities. Indigenous Data Sovereignty remains insufficiently recognised by data custodians.
Lack of expertise	The size and complexity of linked datasets can pose methodological and computational challenges for researchers, requiring substantial technical expertise and resources. There is often a lack of documentation or training on how to effectively use these datasets.
Barriers to collaboration	Lack of clear mechanisms for sharing code, practices and resources limits collaboration and slows progress in using linked administrative datasets effectively.

**TABLE 2:** Summary of common limitations of linked administrative data and corresponding policy implications for child health equity, with examples drawn from work using a longitudinal child‐centred PLIDA data asset [14, 15].

Limitation	Examples	Policy implications
Lack of longitudinal data and data lags	One‐time collection (e.g., parent education, child development outcomes); Census indicators (e.g., housing) updated every 5 years.	Hinders tracking of disadvantage and intervention effects; delays detection of emerging trends; weakens long‐term policy planning.
Gaps in population coverage and representation	Limited indicators for marginalised groups (negatively racialised, First Nations, disability, LGBTIQA+, refugee/asylum seeker, out‐of‐home care, incarcerated); excludes those outside government systems; Indigenous self‐identification may be incomplete due to mistrust or systemic issues.	Undermines representativeness; risks inequitable policies; constrains inclusive, equity‐focused responses.
Challenges tracking mobility and place‐based disadvantage	Outdated or inconsistent addresses; limited neighbourhood indicators (e.g., service access, homelessness).	Obscures geographic disadvantage and mobility; hampers place‐based and spatially targeted policy.
High rates of missing data	High rates of missing data for key indicators (e.g., parent education and occupation, childcare quality).	Weakens data reliability and robustness of evaluation; limits disparity monitoring; risks misinformed policies.
Challenges defining family and household structures	No standardised method to define family composition, co‐parenting or multigenerational households; inconsistent household measures.	Limits understanding of family influence and intergenerational disadvantage; complicates eligibility for family supports.
Reliance on parent or teacher report	Indicators such as home reading or housing conditions based on self‐ or proxy‐report.	Subjectivity and non‐response may underestimate inequities and skew population trends.
Key data not available	Poor capture of social determinants and outcomes (e.g., racism, home environment, caregiver mental health, child wellbeing).	Evidence gaps weaken policy responses; constrain identification of effective levers and long‐term tracking.

Abbreviations: LGBTIQA+, lesbian, gay, bisexual, transgender, intersex, queer, sexual and other sexual or gender diverse; PLIDA, Person Level Integrated Data Asset.

**FIGURE 1:**
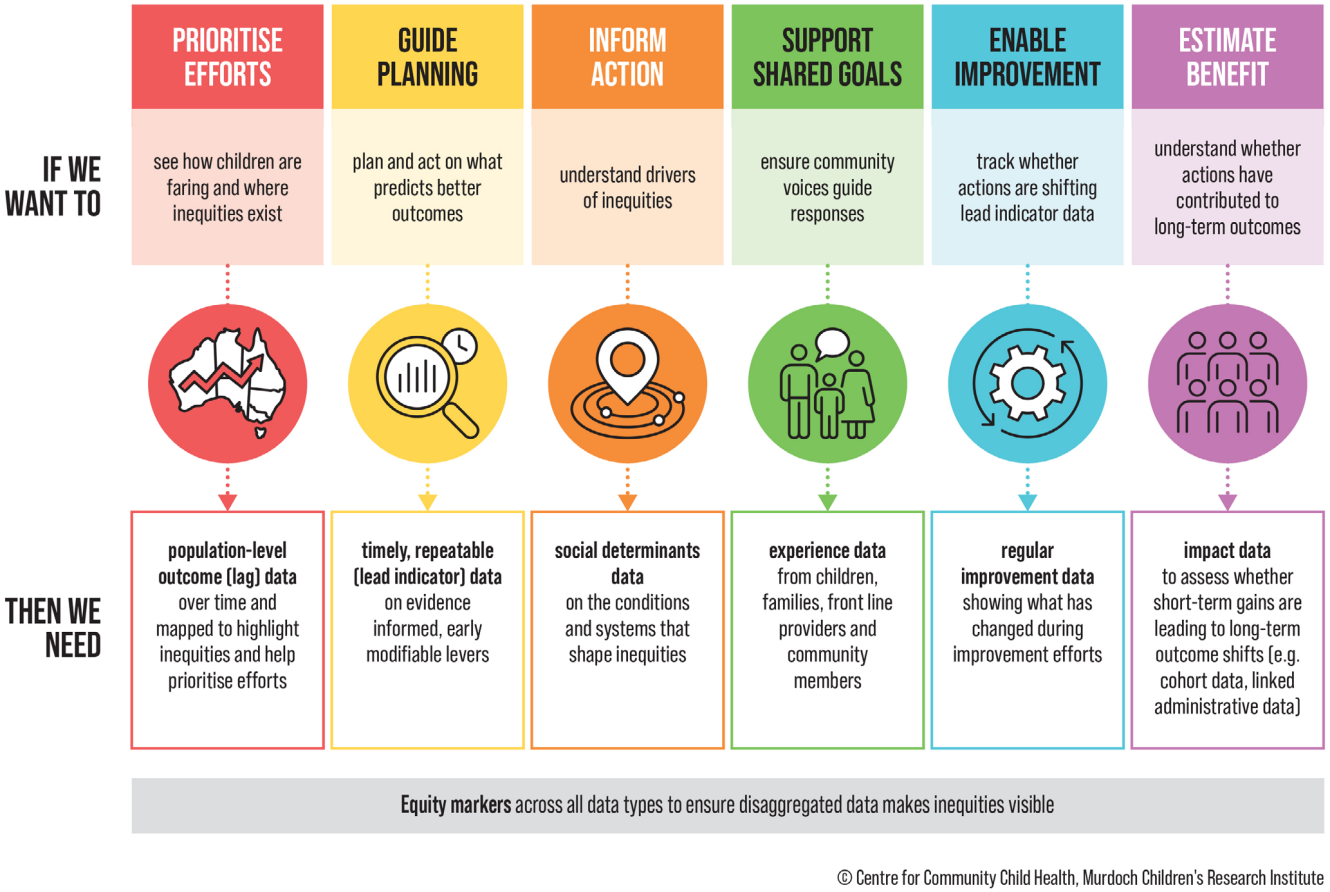
Data logic model linking Australia's data landscape with child equity policy goals. *Source:* Adapted from O'Connor et al. [17].

Policy decisions often rely on long‐term *lag* or outcome indicators, such as disease prevalence or academic achievement [16]. Equally important are *lead* indicators (e.g., access to health care, quality early education), which reveal intervention points on the causal pathway to child development [16]. For equity‐focused action, both must be timely and capture the experiences of priority populations, yet many administrative datasets under‐represent these groups and lack key measures such as ethnicity and racism. An adapted data logic model (Figure 1) linking *lead* and *lag* indicators to equity‐focused policy goals—clarifying what data are needed, for what purposes, and where gaps remain—can help drive lasting improvements in child health [17].

## 3 Turning Data Into Evidence

Turning data into actionable evidence is a key challenge. Causal evidence is needed to identify which interventions can reduce equity gaps, for whom and under what conditions [1]. When paired with robust causal methods, observational data allow exploration of hypothetical cross‐sector policy scenarios that are infeasible, costly or unethical to test in real life [1]. Two approaches are relevant: (i) advanced causal mediation techniques (e.g., interventional effects approach [18]) to estimate how hypothetical interventions on *lead* indicators could improve equity in long‐term outcomes; and (ii) the target trial framework [19], which guides the application of causal inference to observational data by considering how best to emulate an ideal randomised controlled trial and enabling systematic consideration of biases (e.g., measurement, selection, confounding).

Using causal inference methods applied to longitudinal cohort data, evidence suggests that intervening on early, modifiable *lead* indicators (e.g., parent mental health, pre‐school attendance) [20] could yield meaningful reductions in socio‐economic inequities in children's outcomes. Extending these approaches to large‐scale linked administrative data enables testing hypothetical interventions in the populations to whom policies apply, including priority populations often under‐represented in cohorts and traditional trials [21]. Such analyses require technical expertise and careful consideration of social, cultural and structural determinants to ensure findings are rigorous and ethically grounded.

## 4 Evidence to Action

Meaningful impact requires evidence to be co‐designed with policymakers, service leaders and those with lived experience to ensure cultural safety and policy relevance. For priority populations, this approach builds trust and avoids deficit narratives that can reinforce systemic racism or pathologise communities, instead recognising the structural determinants of inequity. For Indigenous communities, co‐design must also uphold Indigenous Data Sovereignty [22], affirming Indigenous peoples' rights to lead governance of data across the lifecycle, from collection to use, in ways that reflect their priorities, values and worldviews.

Deliberate pipelines connecting evidence to timely action are also needed. The COVID‐19 pandemic showed what is possible when routine administrative data (e.g., capturing infection, hospitalisation, vaccination) are rapidly captured, linked and shared to guide timely public health responses [23]. However, translating evidence into policies that narrow equity gaps is rarely linear; change is incremental, shaped by multiple evidence sources and dependent on multi‐portfolio, system‐level action [24]. Without translation structures, research risks becoming extractive, eroding trust and failing to deliver tangible benefits to the populations whose data are used.

## 5 A Path Forward

Analogous to the Australian Synchrotron—a shared infrastructure driving multidisciplinary scientific innovation (https://www.ansto.gov.au/facilities/australian‐synchrotron)—a national linked administrative data system should function as a centralised resource, maximising utility through a ‘collect once, use many times’ approach. This requires investment in key enablers to turn data into evidence and impact:

- *Embed co‐design*: Involve communities, services and policymakers in setting priorities and guiding analyses so evidence reflects real‐world needs.
- *Uphold Indigenous Data Sovereignty*: Address systemic barriers and entrenched power structures, and ensure data use reflects Indigenous rights, priorities and self‐determination [22].
- *Improve equity indicators*: Routinely capture and standardise equity stratifiers (e.g., PROGRESS‐Plus [25]: place of residence, ethnicity, occupation, gender, religion, education, socio‐economic status, social capital, disability) in partnership with the groups they represent.
- *Democratise data governance*: Enable communities, researchers, practitioners and service leaders to participate in data decisions and generate insights and solutions.
- *Enable timely, integrated data*: Remove unnecessary legal and procedural barriers, keep datasets current, securely link across sectors and add new datasets as needed while safeguarding privacy.
- *Bridge data, research and policy*: Establish dedicated translation roles to support evidence use, share methods and build cross‐sector capability, such as embedded researcher positions [26].
- *Continuously improve data*: Embed feedback loops to data custodians to address data quality and equity gaps as research, policy and community needs evolve.

Many of these enablers are already emerging. Internationally, Bradford, United Kingdom (UK), offers a model of co‐design and community governance, demonstrating how communities can guide the use of linked data (Box 1) [27]. In Australia, initiatives such as PLIDA are improving accessibility, expanding data linkages and building a community of practice [7], while the Population Health Research Network (PHRN; https://www.phrn.org.au/) is developing a national metadata platform to support dataset discovery [28]. The inclusion of gender identity and sexual orientation in the 2026 Census shows progress toward better representation of diverse populations [29]. However, these developments must be matched by technical expertise and strong governance to ensure data are used safely and ethically. Debates over potential unethical secondary uses of the UK Biobank, a genetic and health resource of more than 500,000 UK volunteers, highlight the importance of safeguards to ensure that data serve the public good [30].

BOX 1 **Bradford, the city of research [27].**

**Table:** 

Bradford, a diverse city in northern England, exemplifies how linking rich cohort and administrative data with strong governance and community partnership can inform policy to reduce child health inequities. The Born in Bradford (BiB) programme (www.borninbradford.nhs.uk) consists of three birth cohort studies, which have recruited over 30,000 families. These data combine routinely collected information on health, education, social care and environmental factors, complemented by in‐depth research data on the social determinants of health, creating a rich longitudinal resource. Building on BiB, the Connected Bradford programme (https://bradfordresearch.nhs.uk/connected‐bradford/connected‐bradford‐datasets/) now securely links pseudonymised health, education, social care, housing and environmental data for all Bradford residents, creating one of the United Kingdom's most comprehensive whole‐population health datasets. Bradford's data infrastructure is governed and shaped by local services, policymakers and communities who co‐produce research priorities, help interpret findings and co‐create solutions, ensuring that evidence translates into practical action. This City of Research approach has revealed key drivers of inequities in contemporary urban populations. For example, identifying stark inequalities in the uptake of early years services, as well as poorer developmental, health and education outcomes among children from some ethnic minority groups and families experiencing disadvantage. These insights have informed tailored community health initiatives, targeted early years programmes and environmental health strategies to address local inequities. Bradford's experience demonstrates the power of harmonising cohort and administrative data, underpinned by strong governance and community trust, to generate actionable insights for more equitable child health policy—highlighting practical lessons and potential rewards for Australia's efforts to build a national data infrastructure to support precise, equitable policy.

Administrative data alone cannot generate the evidence required to meaningfully advance equity. Longitudinal cohorts such as the Longitudinal Study of Australian Children (LSAC) [31] and ORIGINS [32] capture early‐life environments not available in administrative data, while Generation Victoria (GenV) [33] with over 120,000 participants, shows how cohorts can function as population‐scale research infrastructure. Integrating administrative and cohort data already offers powerful opportunities for policy‐relevant evidence, with Bradford illustrating how this can drive impact when underpinned by strong governance (Box 1) [27]. A coordinated approach to harmonising these data sources and leveraging their complementary strengths is now needed. The UK's Longitudinal Linkage Collaboration (LLC) provides one model, linking over 20 cohort studies with cross‐sector administrative data and harmonising core variables to support pooled, policy‐relevant analyses [34].

## 6 Conclusion

Data‐driven pathways to more equitable child health are achievable. We need a national children's data strategy to harmonise data that can generate timely evidence and inform responsive policy. This must bring together federal and state governments, researchers, data custodians and communities, supported by shared investment and governance to ensure data are translated into impact.

## Author Contributions

**Sarah Gray:** conceptualisation, investigation, methodology, project administration, visualisation, writing (original draft), writing (review and editing); **Shuaijun Guo:** conceptualisation, investigation, writing (review and editing); **Meredith O'Connor:** conceptualisation, writing (review and editing); **Elodie O'Connor:** conceptualisation, visualisation, writing (review and editing); **Katrina Williams:** writing (review and editing); **Hannah Badland:** writing (review and editing); **Susan Woolfenden:** writing (review and editing); **Josie Dickerson:** writing (review and editing); **Gerry Redmond:** writing (review and editing); **Marnie Downes:** writing (review and editing); **Sharon R. Goldfeld:** conceptualisation, methodology, supervision, writing (review and editing).

## Funding

This work was supported by the Australian Research Council (ARC) Linkage Projects (LP190100921), the Australian National Health and Medical Research Council (NHMRC) Partnership Project Grant (APP 2040703) and the Victorian Government's Operational Infrastructure Support Program. Prof Goldfeld is supported by the NHMRC 2023 Investigator Grant (2026263). Hannah Badland is supported by an ARC Future Fellowship (FT230100131). The funding sources had no role in the planning, writing or publication of the work.

## Disclosure

Not commissioned; externally peer reviewed.

## Conflicts of Interest

The authors declare no conflicts of interest.

## Data Availability

The authors have nothing to report.
